# LncRNA H19 Promotes Gastric Cancer Metastasis via miR-148-3p/SOX-12 Axis

**DOI:** 10.1155/2024/6217134

**Published:** 2024-08-17

**Authors:** Xin Zhang, Ge Wang, Xiaoru Li, Yanqing Liu, Xue Wu, Yazhe Zhou, Jie Liu, Haiying Wang, Rui Jiao, Ying Chen, Qiang Wang

**Affiliations:** ^1^ Department of Orthopedics Shenmu Hospital Faculty of Life Sciences and Medicine Northwest University, Guangming Road, Shenmu 719300, China; ^2^ Department of Cardiology Affiliated Hospital Yan'an University, 43 North Street, Yan'an 716000, China; ^3^ Department of Cardiovascular Surgery Guangdong Provincial Hospital of Chinese Medicine The Second Affiliated Hospital of Guangzhou University of Chinese Medicine, Guangzhou 510405, China; ^4^ Key Laboratory of Resource Biology and Biotechnology in Western China Ministry of Education Faculty of Life Sciences and Medicine Northwest University, 229 Taibai North Road, Xi'an 710069, China; ^5^ Department of Oncological Surgery Shenmu Hospital Faculty of Life Sciences and Medicine Northwest University, Guangming Road, Shenmu 719300, China; ^6^ Department of Hematology The First Affiliated Hospital of Xi'an Jiaotong University, 277 Yanta West Road, Xi'an 710061, China

## Abstract

**Background:**

Gastric cancer (GC) is the most common malignant tumor and ranks third in the world. LncRNA H19 (H19), one of the members of lncRNA, is overexpressed in various tumors. However, many undetermined molecular mechanisms by which H19 promotes GC progression still need to be further investigated. *Methodology*. A series of experiments was used to confirm the undetermined molecular mechanism including wound healing and transwell assays. *Key Results*. In this study, a significant upregulation of H19 expression was detected in GC cells and tissues. The poor overall survival was observed in GC patient with high H19 expression. Overexpression of H19 promoted the migration of GC cells, while knockdown of H19 significantly inhibited cell migration. Moreover, miR-148a-3p had a certain negative correlation with H19. Luciferase reporter assay confirmed that H19 could directly bind to miR-148a-3p. As expected, miR-148a mimics inhibited cell migration and invasion induced by H19 overexpression. The above findings proved that H19 functions as a miRNA sponge and verified that miR-148a-3p is the H19-associated miRNA in GC. We also confirmed that SOX-12 expression was upregulated in GC patient's samples. SOX-12 expression was positively correlated with expression of H19 and was able to directly bind to miR-148a-3p. Importantly, *in vitro* wound healing assay showed that knockout of SOX-12 could reverse the promoting effect of H19 overexpression on cell migration.

**Conclusion:**

In conclusion, H19 has certain application value in the diagnosis and prognosis of GC. Specifically, H19 accelerates GCs to migration and metastasis by miR-138a-3p/SOX-12 axis.

## 1. Introduction

Gastric cancer (GC) is a common malignant tumor of the digestive system, and its mortality rate ranks third among malignant tumors [1]. Currently, the clinical treatment of GC mainly includes surgery, radiotherapy, and chemotherapy [2]. Despite the great progress made in early diagnosis and treatment of GC, the 5-year survival rate for advanced GC is still unsatisfactory [3]. Therefore, it is imperative to deeply explore the molecular mechanisms of GC and to identify possible therapeutic targets for the diseases.

Accumulated evidence shows that various long non-coding RNAs (lncRNAs) are associated with disorders in tumors, and only a few of these disordered lncRNAs are closely related to predicting cancer prognosis [4]. LncRNAs are an RNAs that are >200 nt in length and no protein-coding function, which participate in various biological events by regulating chromatin, transcription, and posttranscriptional gene expression [5]. LncRNA H19 (H19), one of the members of lncRNA, is overexpressed in various tumors including breast, colorectal, and lung cancer [6]. Importantly, the elevation of H19 in GC can predict poor prognosis of patients, and thus downregulation of H19 expression can suppress the GC cells migration and invasion [7, 8]. Zhang et al. [9] found that H19 level was significantly elevated in GC tissues and was positively correlated with worse overall survival (OS). In addition, H19 has also been shown to play a critical part in regulating migration, growth, and invasion of GC cell [7, 10]. However, the many undetermined molecular mechanisms by which H19 promotes GC progression still need to be further investigated.

In this study, we investigated the expression and regulation of H19 in GC. The results founded that H19 expression was remarkably elevated in GC tissue and correlated with poor prognosis. Notably, H19 accelerates GCs to migration and metastasis by miR-138a-3p/SOX-12 axis.

## 2. Materials and Methods

### 2.1. Human GC Tissues

The 25 clinical GC patients included 16 males (64%) and 9 females (36%). The GC tissues were obtained from surgical patients who originated from Shenmu Hospital affiliated with Northwestern University and gave prior written informed consent (sm008, Table 1). Experimental procedures using human GC tissues conformed to the principles outlined in the Declaration of Helsinki and were performed with the approval by the Ethics Committees of Shenmu Hospital. The histological evaluation was performed by two pathologists in a double-blind manner.

### 2.2. Cell Lines and Treatment

For the specific culture methods of immortalized gastric mucosal epithelial cells (GES-1) and five GC cell lines (MKN-45, BGC-823, MKN-28, AGS, and MGC-803), please refer to the study of Zhang et al. [11]. AGS cell line was transfected with empty vector or H19 overexpressing plasmid to establish H19 overexpressing cell lines. Whereas BGC-823-*sh*H19 cell line was established by lentivirus infections.

Knockdown of human H19 was performed using the lentiviral plasmids pLenti-siH19-GFP (Abcam, #i009382) and pLenti-scrambled siRNA-GFP (Abcam, #LV015-G) was used as a control. These siH19 plasmids allowed for direct nonviral plasmid transfection for immediate expression (siH19) and packaged into lentiviral particles for high-efficiency transduction and stably integrated expression (shH19). Of the four siRNA target sequences we tested, two (shH19-C and shH19-D) demonstrated a functional knockdown:

  shH19-A 1483: GAAGCGGGTCTGTTTCTTTACTTCCTCCA.
  shH19-B 1551: ACCCACAACATGAAAGAAATGGTGCTACC.
  shH19-C 1589: CCTGGGCCTTTGAATCCGGACACAAAACC.
  shH19-D 1710: CCTCATCAGCCCAACATCAAAGACACCAT.

For all experiments, shH19-C was used unless indicated otherwise.

### 2.3. RNA Extraction and PCR

RNA extraction [12], reverse transcription (miR-148-3p) [13], and qRT-PCR (mRNA and lncRNA H19) were mentioned, as described previously [14]. The primers used in this study are shown as follows: H19, Fw: 5′TCCTGAACACCTTAGGCTGG3′; Rev: 5′TGATGTTGGGCTGATGAGGT3′; SOX-12, Fw: 5′GACATGCACAACGCCGAGATCT′; and Rev: 5′GTAATCCGCCATGTGCTTGAGC′.

### 2.4. Luciferase Reporter Assay

HEK-293T cells were seeded (1.0 × 10^6^ cells/well) for 24 hr before transfection. Cells were cotransfected with the miR-148a-3p mimics and SOX-12 wild-type plasmids/mutant-type plasmids or H19 wild-type plasmids/mutant-type plasmids. Then, the cells were incubated for 24 hr and analyzed with a Dual-Luciferase Reporter Gene Assay Kit (Beyotime, China) following the manufacturer's instructions. Please refer to the study of Zhao et al. [15] for specific operation steps.

### 2.5. Transwell Cancer Cell Migration and Invasion Assay

For cell migration assay, a total of 5 × 10^4^ transfected GC cells were placed in the upper chamber. Meanwhile, fresh medium with 20% FBS was added into the lower chamber, which was aimed to induce the migration of GC cells. For cell invasion assay, a total of 5 × 10^4^ transfected GC cells were seeded into 24-well insert transwell invasion chambers (Corning, NY, USA), and the lower chamber contained medium with 20% FBS was added into the lower chamber. Cells without migration were removed with a cotton swab after 24 hr of incubation. The migrated cells were immobilized with methanol, then staining with crystal violet and calculated by microscope (Olympus Corporation, Tokyo, Japan). For a detailed description of the transwell cancer cell migration and invasion assay, please refer to Zhang et al.'s [16] study.

### 2.6. Wound Healing Assay

Transfected GC cells were seeded in a 12-well plate with 80% of confluence. The vertical and a horizontal line was drawn on the bottom of each well by using a sterile pipette tip and cultured for 24 hr. The same view of the scratch was captured, and the migration rate was evaluated by Image J software.

### 2.7. In Vivo Metastasis Model of Nude Mice

Six-week-old male nude mice (*n* = 5 per group) were used for *in vivo* assay. All animal experiments were approved by the Animal Ethics Committee of Northwest University (NWU-AWC-20211001M).

Nude mice were sedated using 2% isoflurane inhalation anesthesia, and the cells of BGC-823-*sh*control and BGC-823-*sh*H19 (a total of 1 × 10^6^/200 *µ*l) were injected into the mice via tail vain. The mice were sacrificed 6 weeks after induction. The lungs were collected, and the number of metastasis nodules was counted according to HE stains.

### 2.8. Statistical Analysis

Data are specified as mean ± standard deviation. Statistical analysis was performed between the two groups using *t*-test. Statistical comparisons among groups were performed using analysis of variance (ANOVA). All analyses data were performed by SPSS 21.0 software (IBM, USA), and the two-tailed value was considered statistically significant (*p* < 0.05).

## 3. Results

### 3.1. H19 Was Highly Expressed in GC Tissues and Cell Lines

**Figure 1:**
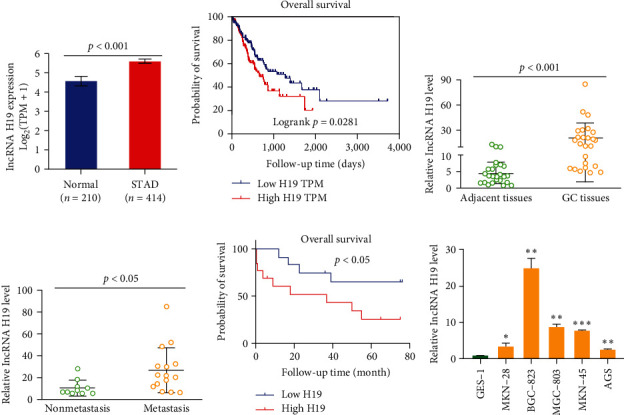
Long non-coding RNAs H19 was highly expressed in GC tissues and cell lines. (a) H19 was highly expressed in GC tissues from TCGA database. (b) High expression of H19 was corrected with poor prognosis in GC tissues. (c) H19 was highly expressed in GC patients tissues, *n* = 25. (d) H19 was higher in GC metastatic tissues than in nonmetastasis GC tissues. (e) High H19 expression was observed poor OS in GC clinical samples. (f) H19 was highly expressed in five human GC cell lines (MKN-28, BGC-823, MGC-803, MKN-45, and AGS).  ^*∗*^*p*  < 0.05 level,  ^*∗∗*^*p*  < 0.01, and  ^*∗∗∗*^*p*  < 0.001.

To investigate the level and OS of H19 in GC, we analyzed 210 normal and 414 GC tissues in The Cancer Genome Atlas (TCGA-GTEx) database. As shown in Figures 1(a)and 1(b), H19 was overexpressed in the GC tissues, and the poor OS was observed in GC patient with high H19 expression (vs. normal group). We then examined H19 expression in 25 clinical GC samples and their matched adjacent tissues. Compared with adjacent tissues, H19 was highly expressed in GC tissues. Furthermore, H19 was higher in GC metastatic tissues than in nonmetastasis GC tissues, suggesting that elevated H19 expression may participate in the progression of GC (Figures 1(c) and 1(d), *p* < 0.05). More importantly, poor OS was observed in GC clinical samples with high H19 expression (Figure 1(e), *p* < 0.05). In addition, we determined H19 levels in GC cell lines widely used in piles of research, including MKN-28, BGC-823, MGC-803, MKN-45, and AGS. We found that all the GC cell lines showed increased expression of H19 compared with normal GES. Based on the background level of H19, we selected BGC-823 cell line, which showed the highest H19 level, to construct H19 downregulation cell lines. Accordingly, we overexpressed H19 in AGS cell line to perform further study (vs. GES cell, Figure 1(f), *p* < 0.05).

### 3.2. H19 Was Associated with the Metastasis Ability of GC In Vitro and In Vivo

**Figure 2:**
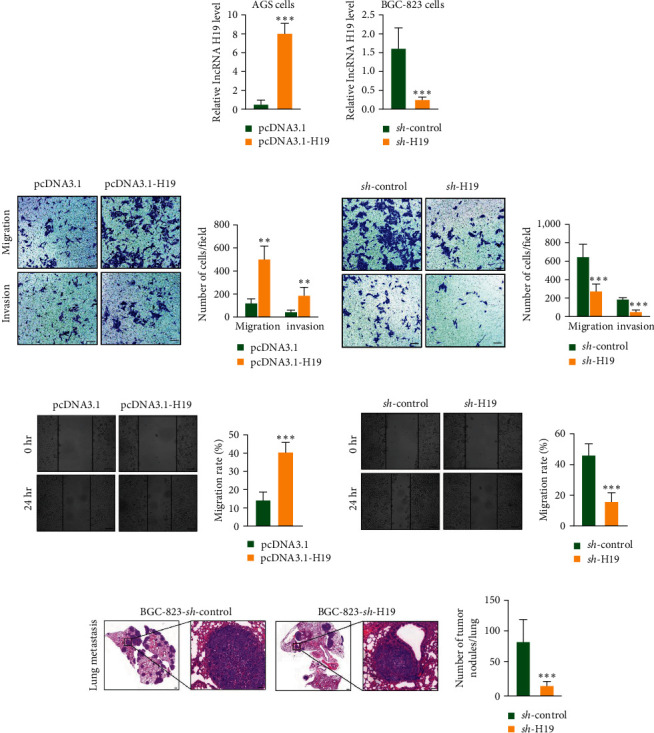
Long non-coding RNAs H19 was associated with the metastasis ability of GC *in vitro* and *in vivo*. (a) Overexpression or knockdown of H19 was constructed with AGS or BGC-823 cells. (b) Transwell assays confirmed that H19 overexpression accelerated cell migration and invasion. Knockdown of H19 suppressed cell migration and invasion. Scale bars, 10 *µ*m. (c) Wound healing assay proved that H19 overexpression accelerated metastasis of GC cells. H19 knockdown suppressed metastasis of GC cells. Scale bars, 10 *µ*m. (d) Knockdown of H19 inhibited the migration of GC in a metastasis nude mouse model. Scale bars, 100 *µ*m.  ^*∗∗*^*p*  < 0.01 and  ^*∗∗∗*^*p*  < 0.001.

As mentioned above, BGC-823 cells showed the greatest increase in expression of H19, and AGS cells showed the lowest expression of H19. Therefore, in BGC-823 cells, we constructed the lentiviral shRNA vector targeting H19 to generate sh-H19. AGS cells were nucleotransfected in the presence of H19 (H19-pcDNA3.1) or empty vector (pcDNA3.1), which was used to construct overexpression H19 (H19-OE). Successful overexpression and knockdown of mRNA expression was confirmed with RT-qPCR analysis (Figure 2(a)). The migration of GC cells was enhanced by H19 overexpression, while knockdown of H19 dramatically inhibited the GC cells migration using wound healing and transwell assays (Figures 2(b) and 2(c)). To further confirm these findings, we established a metastasis model in nude mice by tail vein injection of BGC-823 cells. As expected, sh-H19 also inhibited the migration of GC *in vivo* (Figure 2(d)) [17, 18].

### 3.3. miR-148a-3p Was Low Expressed in GC Cell Lines

**Figure 3:**
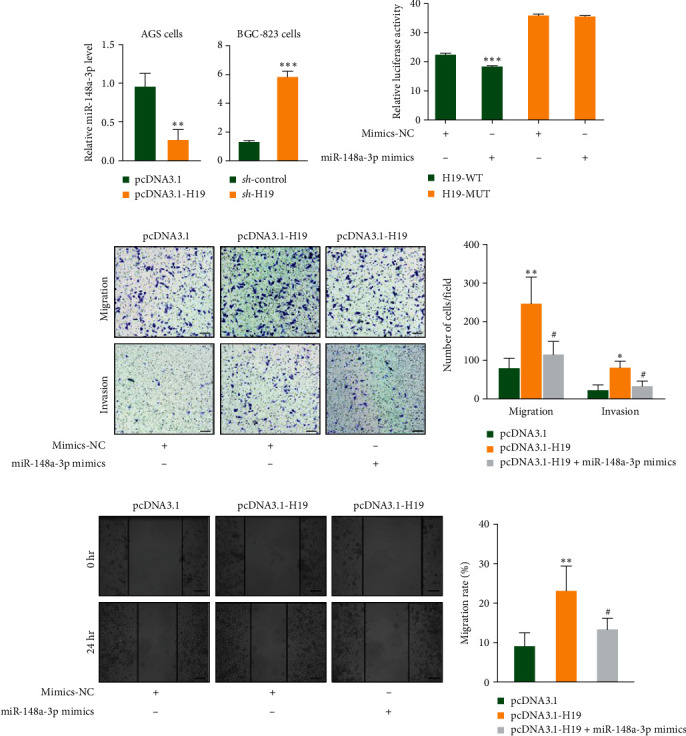
miR-148a-3p was low expressed in GC cell lines. (a) miR-148a-3p was downregulated in H19 overexpression cell lines and upregulated in H19 knockdown cell lines. (b) Luciferase reporter assays confirmed that H19 could directly bind to miR-148a-3p. (c) Transwell analysis showed miR-148a-3p mimic suppressed migration and invasion of GC cells induced by H19 overexpression. (d) Wound healing assay verified miR-148a-3p mimic suppressed migration of GC cells induced by H19 overexpression. Scale bars, 10 *µ*m.  ^*∗*^*p* < 0.05,  ^*∗∗*^*p* < 0.01 (pcDNA3.1-H19 vs. pcDNA3.1), and ^#^*p*  < 0.05 (pcDNA3.1-H19 + miR-148a-3p mimics vs. pcDNA3.1-H19).  ^*∗∗∗*^*P* < 0.001.

Studies have found that H19 acted as a tumor promoter gene in multiple cancer by modulating miR-148a-3p signaling pathway [19, 20]. As shown in Figure 3(a), miR-148a-3p was descended in H19 overexpression cell lines and upregulated in H19 knockdown cell lines, suggesting that there may be a negative correlation between H19 and miR-148a-3p. Then, H19 could directly bind to miR-148a-3p by using luciferase reporter assays (Figure 3(b)). Subsequently, we transfected miR-148a-3p mimics into AGS cells, to explore the biological function of miR-148a-3p in GC cells. The results, as shown in Figure 3(c), indicated that miR-148a mimic suppressed cell migration and invasion induced by H19 overexpression. Moreover, miR-148a-3p mimic also suppressed GC migration effect of H19 overexpression by using wound healing assay (Figure 3(d)). These results of this study strongly support the view that H19 can act as a miRNA sponges and suggest that miR-148a-3p is an H19-associated miRNAs in GC.

### 3.4. H19 Acted as a Sponge of miR-148a-3p that Directly Targeted SOX-12

**Figure 4:**
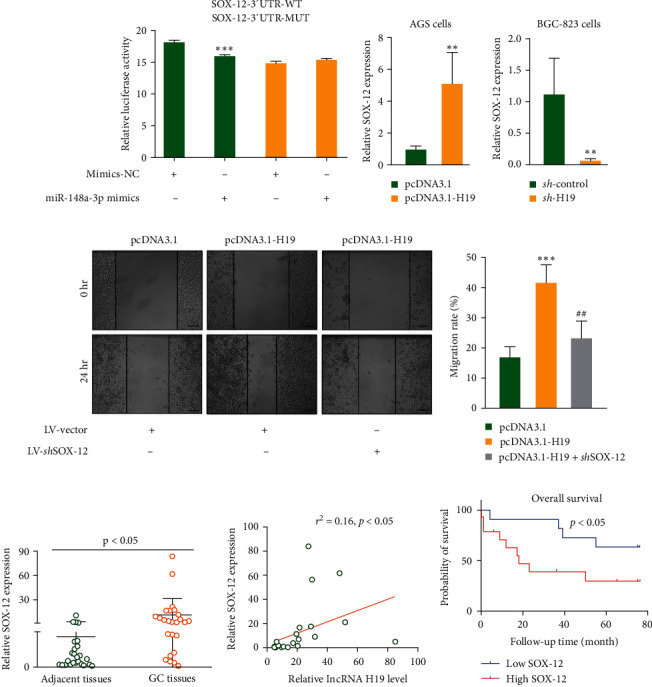
Long non-coding RNAs H19 acted as a sponge of miR-148a-3p that directly targeted SOX-12. (a) Luciferase reporter assays confirmed that miR-148a-3p could directly bind to SOX-12. (b) SOX-12 was upregulated in H19 overexpression cell lines and downregulated in H19 knockdown cell lines. (c) Wound healing assay showed shSOX-12 inhibited migration of GC cells induced by H19 overexpression. Scale bars, 10 *µ*m.  ^*∗∗∗*^*p* < 0.001 (pcDNA3.1-H19 vs. pcDNA3.1) and ^##^*p*  < 0.01 level (pcDNA3.1-H19+*sh*SOX-12 vs. pcDNA3.1-H19). (d) SOX-12 was highly expressed in cancer tissues from GC patients, *n* = 25. (e) H19 expression was positively correlated with SOX-12 expression in clinical GC sample. (f) High SOX-12 predicted poor prognosis in GC patients.  ^*∗∗*^*p*  < 0.01 and  ^*∗∗∗*^*p*  < 0.001.

SOX-12 is overexpressed in GC and correlates with poor prognosis, which plays a vital role in GC progression and metastasis [17, 21]. To further affirm whether SOX-12 is a direct target of miR-148a-3p, luciferase reporter assay was carried out. These results indicate that SOX-12 could directly bind to miR-148a-3p (Figure 4(a)). Furthermore, knockdown of H19 downregulated SOX-12 while overexpression of H19 upregulated it in BGC-823 and AGS cells, respectively (Figure 4(b)). Importantly, *in vitro* wound healing assay confirmed that knockout of SOX-12 could reverse the promoting effect of H19 overexpression on cell migration (Figure 4(c)). Similarly, SOX-12 expression was upregulated in GC patient's samples (Figure 4(d)). In addition, SOX-12 expression was positively correlated with expression of H19 (Figure 4(e)). More importantly, high levels of SOX-12 predicted for poor survival in GC clinical samples (Figure 4(f)).

## 4. Discussion

Late detection and poor prognosis are the main reasons that threaten GC patients [22]. Although some progress has been made in GC development, the prognosis of patients with GC remains unfavorable [23]. LncRNAs (longer than 200 nt) have limited protein-coding capacity, exert essential effects in cancer progression and metastasis [24]. LncRNA-based clinical tools are promptly evolving, including diagnostic, prognostic biomarkers, and therapeutic targets [25, 26]. Previous studies found the function of H19 in multiple tumor types, but H19 expression in GC and its clinical significance remain unclear [27, 28].

LncRNAs have important biological functions, and H19 is one of the first lncRNA to be discovered [29]. The gene encoding H19 is located in the imprinted region of chromosome 11 near the insulin-like growth factor 2 (IGF2) gene, and several transcripts are encoded from the H19/IGF2 locus [30]. During embryonic development, H19 is highly expression and is repressed in most tissues after birth [31]. A study linking H19 to cancer reported that H19 was elevated in bladder cancer and considered it a predictor of early cancer recurrence [32]. Since then, H19 has been observed to be overexpressed in many tumors, including GC, and plays an essential role in cancer progression and metastasis [33]. Elevated levels of H19 have also been examined in the plasma of GC patients as a potential diagnostic marker [34]. In this study, evidently, upregulated expression of H19 was detected in GC cells and tissues. The poor OS was observed in GC patient with high H19 expression. Moreover, the migration of GC cells was enhanced by H19 overexpression, while knockdown of H19 dramatically inhibited the GC cells migration by using wound healing and transwell assays.

Assessment of H19 on cell migration and invasion is a key determinant of malignant progression and metastasis [35]. Liu et al. [36] confirmed the promotion of H19 on GC cell epithelial–mesenchymal transition (EMT) and metastasis *in vitro* and *in vivo*. Mechanistically, H19 could induce *β*-catenin to transfer into nucleus and activate Wnt/*β*-catenin signaling, thus promoting EMT and metastasis of GC cells [36]. Gan et al. [37] also verified that the downregulation of H19 suppressed the proliferation, invasion, migration, and EMT of GC cells *in vitro* and suppressed tumor growth *in vivo*. Moreover, multiple well-established research used to mimic the metastasis *in vivo*, which is consistent with our study [35, 38, 39, 40]. In this study, we found that H19 was associated with the metastasis ability of GC *in vitro* and *in vivo*. The migration of GC cells was enhanced by H19 overexpression, while knockdown of H19 dramatically inhibited the GC cells migration. Subsequently, we established a metastasis model in nude mice by tail vein injection of BGC-823 cells. As expected, sh-H19 also inhibited the migration of GC *in vivo*.

miRNAs, as small non-coding RNAs of about 22 nt, have potential roles in the modulation of gene level at posttranscriptional level [41]. Growing evidence has elucidated the key roles of aberrant miRNAs in regulating carcinogenesis [42]. miR-148a-3p is an important regulator of multiple cancer (including pancreatic, breast, and lung cancer) via modulating cell proliferation and apoptosis [42]. A study confirmed that miR-148a-3p modulates MEG3 through targeting DNA methyl transferase 1 in GC [43]. Moreover, miR-148a-3p can also inhibit GC cell invasion and migration and is associated with 5-year disease-free survival [44]. Moreover, miR-148a is indeed a member of the miR family, which plays a crucial role in regulating gene expression posttranscriptionally. In GC, miR-148a, along with its family member's miR-148b and miR-152, has been found to be dysregulated, indicating its potential significance in the pathogenesis of this disease. Notably, miR-148a, miR-148b, and miR-152 share the same seed sequence. The seed sequence is a crucial region within the miRNA molecule that determines its target specificity. Despite sharing this sequence, miRNAs can still have distinct functions due to differences in other regions of their sequence or their expression patterns. The dysregulation of miR-148a, along with its family members, in GC underscores the complex regulatory networks involved in cancer development and progression. In this study, among various miRs that predicted could interact with H19, miR-148a showed the most significant correlate with H19, thus we focused on miR-148a [45, 46]. In this study, H19 may be negatively correlated with miR-148a-3p, and H19 could directly bind to miR-148a-3p. Furthermore, miR-148a mimics inhibited cell migration and invasion induced by H19 overexpression. The above findings proved that H19 functions as a miRNA sponge and verified that miR-148a-3p is the H19-associated miRNA in GC.

The transcription factor SOX-12 belongs to SOX family, which participates in maintaining the pluripotency, self-renewal, and differentiation of embryonic stem cells [47]. In hepatocellular carcinoma cells, overexpression of SOX-12 can induce EMT, invasion, and metastasis, which is also considered to be a potentially promising target for this disease [48]. SOX-12 has also been confirmed to be participate in the progression of leukemia via modulation of *β*-catenin expression and interference of TGF/Wnt pathway [49]. Furthermore, knockdown of SOX-12 expression suppresses the growth, migration, and invasion of lung and breast cancer cells [50, 51]. Notably, Du et al. [17] found that overexpression of SOX-12 promoted GC cell migration, invasion, and metastasis, whereas SOX-12 downregulation reversed these effects. These studies suggest that SOX-12 plays an essential role in tumor progression and metastasis. However, the effect of SOX-12 in human GC with H19 and miR-148a-3p has not been clarified. In this study, SOX-12 expression was upregulated in GC patient's samples. In addition, SOX-12 expression was positively correlated with expression of H19, and SOX-12 could directly bind to miR-148a-3p. Importantly, *in vitro* wound healing assay confirmed that knockout of SOX-12 could reverse the promoting effect of H19 overexpression on cell migration. These results suggest that SOX-12 may be a therapeutic target and a prognostic marker for GC. However, due to the small sample size in this study and the large individual variation in GC patients, the differences were not satisfactory (but were statistically significant), which is also a limitation of this study and will hopefully be explored in depth in further studies.

## 5. Conclusion

In summary, these findings provide novel insight into the potential regulatory roles of H19 in GC and suggest that the H19/miR-148a-3p/SOX-12 axis may prove to be a promising therapeutic target for the treatment of patients with GC.

## Figures and Tables

**Table 1:** Clinical characteristics of gastric cancer patients.

Characteristics	*n*
Total cases	25
Gender	
Male	16
Female	9
Age (years)	
≤60	15
>60	10
Smoking history	
Yes	7
No	18
Tumor location	
Cardia of stomach	7
Fundus of stomach	2
Gastric body	4
Antrum of stomach	12
Tumor diameter (cm)	
≤3	17
>3	8
Differentiation	
High and moderate	12
Poor	13
TNM stage	
Ⅰ	4
Ⅱ	7
Ⅲ and Ⅳ	14
Histological type	
Ulcerative type	25

## Data Availability

The raw data supporting the conclusions of this article will be made available by the authors, without undue reservation. The datasets used and/or analyzed in the current study are available from the corresponding author upon reasonable request.

## Abbreviations

GC: Gastric cancer
LncRNAs: Long non-coding RNAs
H19: LncRNA H19
OS: Overall survival
GES-1: Gastric mucosal epithelial cells
TCGA: The cancer genome atlas
IGF2: Insulin-like growth factor 2.
